# Comparative analysis of respiratory metabolism, blood physiology, antioxidant capacity, and hypoxia-related gene expression in Snowy White chickens raised at high and low altitudes

**DOI:** 10.1016/j.psj.2025.105378

**Published:** 2025-05-30

**Authors:** Yang Liu, Felix Kwame Amevor, Kunlong Qi, Jing Feng, Lili Xian, Zang Lei, Leilei Peng, Dan Xu, Gang Shu, Yingjie Wang, Liuting Wu, Yan Wang, Xiaoling Zhao

**Affiliations:** a State Key Laboratory of Swine and Poultry Breeding Industry, College of Animal Science and Technology, Sichuan Agricultural University, Chengdu, Sichuan, 611130, PR China; b Farm Animal Genetic Resources Exploration and Innovation Key Laboratory of Sichuan Province, College of Animal Science and Technology, Sichuan Agricultural University, Chengdu, Sichuan, 611130, PR China; c Key Laboratory of Livestock and Poultry Multi-omics, Ministry of Agriculture and Rural Affairs, Sichuan Agricultural University, Chengdu, Sichuan, 6611130, PR China; d Institute of Animal Husbandry and Veterinary Medicine, College of Agriculture and Animal Husbandry, Tibet Autonomous Region, Lhasa 850004; e Department of Basic Veterinary Medicine, Sichuan Agricultural University, Chengdu, Sichuan, 611130, PR China

**Keywords:** Altitude adaptation, Oxidative stress biomarker, Hypoxic gene response, Poultry physiology, Snowy White chicken

## Abstract

Understanding the physiological and molecular mechanisms underlying altitude adaptation is critical for optimizing poultry health and performance in diverse environments. This study comparatively evaluated the respiratory metabolism, hematological profiles, antioxidant capacity, immune status, and hypoxia-related gene expression in Snowy White chickens raised at contrasting altitudes. A total of 380 chickens from a low-altitude region (Sichuan) and 550 from a high-altitude region (Tibet) were reared under standardized dietary and management conditions. The results should that key indicators of anaerobic metabolism, including serum lactate and lactate dehydrogenase (LDH) levels, were significantly elevated in high-altitude chickens, indicating greater reliance on glycolysis under hypoxic stress (*P* < 0.05). Hematological analysis revealed significantly increased red blood cell (RBC) count, hemoglobin concentration, hematocrit, and mean corpuscular hemoglobin in high-altitude birds (*P* < 0.05), while mean corpuscular volume (MCV) was higher in low-altitude chickens (*P* < 0.05), reflecting divergent strategies in oxygen transport efficiency. Oxidative stress markers showed that high-altitude chickens had elevated malondialdehyde (MDA) levels, indicating increased lipid peroxidation, whereas low-altitude chickens demonstrated superior antioxidant defense, with significantly higher total antioxidant capacity (T-AOC) and total superoxide dismutase (T-SOD) activity (*P* < 0.05). In addition, immunoglobulin levels (IgM and IgG) were markedly higher in low-altitude chickens, suggesting enhanced immune responsiveness. Furthermore, qRT-PCR revealed elevated expression of hypoxia-related genes including hypoxia-inducible factor 1-alpha *(HIF-1A),* endothelial PAS domain-containing protein 1 *(EPAS1),* vascular endothelial growth factor *(VEGF),* and erythropoietin *(EPO)* in the heart, lungs, and kidneys of the high-altitude chickens (*P* < 0.05), while egl-9 family hypoxia-inducible factor 1 *(EGLN1)* expression was significantly downregulated in these tissues. These physiological and molecular adaptations highlight the mechanisms by which Snowy White chickens maintain homeostasis under chronic hypoxic stress and offer insight into genetic and metabolic pathways supporting high-altitude resilience. Taken together, these findings offer valuable insights into high-altitude resilience in avian species and may inform breeding strategies for improved adaptability to hypoxic environments.

## Introduction

High-altitude environments are characterized by low atmospheric pressure and oxygen levels, which impose significant stress on animal organisms. To cope with environmental challenges, animals have developed various adaptive mechanisms (Li et al., 2021). Under normal conditions, they primarily rely on aerobic respiration for energy production. However, in low-oxygen, high-altitude environments, anaerobic respiration is activated as a compensatory pathway to counter reduced oxygen availability. This physiological adaptation results in alterations in respiratory enzyme activities, enabling animals to utilize available oxygen more efficiently for energy metabolism (Gu et al., 2024; Jia et al., 2016; Tang et al., 2021). Blood physiology also undergoes significant adjustments in response to hypoxic conditions (Yan et al., 2024; Yu et al., 2024). Erythrocytes, the most abundant blood cells, play a crucial role in oxygen transport throughout the body. Hemoglobin in red blood cells binds to oxygen in the lungs and delivers it to tissues (Richardson et al., 2020). In high-altitude environments, animals adapt by increasing red blood cell count and hemoglobin concentration to boost oxygen-carrying capacity; however, this compensatory response may also result in elevated blood viscosity and increased blood pressure (Okur et al., 2022; Yu et al., 2024; Zhu et al., 2020). Oxidative stress is another major consequence of high-altitude living (Pena et al., 2022). In hypoxic conditions, cells generate elevated levels of reactive oxygen species (ROS), which can disrupt the balance between oxidative and antioxidative processes, leading to oxidative stress (Gaur et al., 2021; Pena et al., 2022; Siques et al., 2018; Soliman et al., 2022).

Hypoxia-related genes, such as EGLN1, play a pivotal role in the body’s response to low oxygen. Under normal oxygen conditions, EGLN1 hydroxylates and degrades hypoxia-inducible factors (HIFs), including HIF-1A and EPAS1(Shams et al., 2023; Zhang et al., 2019). However, during hypoxia, EGLN1 activity is inhibited, stabilizing the expression of HIF-1A and EPAS1 (Shams, et al., 2023; Zhang, et al., 2019). These transcription factors activate the expression of downstream genes like VEGF and EPO (Vageli et al., 2024; Yang et al., 2022), which contribute to angiogenesis, increased blood flow to tissues, and enhanced erythropoiesis to improve oxygen transport (Suzuki et al., 2024; Wang et al., 2024). These mechanisms ensure that animals in low-oxygen environments can meet their increased oxygen and nutrient demands for growth and survival.

The Snowy White chicken, a high-quality lightweight egg-laying breed developed in the Tibet Autonomous Region, is known for its strong adaptability to high-altitude environments. Native to Lhasa, Tibet, at an altitude of 3,650 meters, this breed thrives in low-oxygen conditions. However, after being introduced to a lower-altitude environment in Ya'an, Sichuan Province, at approximately 622 meters, where oxygen availability is higher, differences in respiratory metabolism, oxidative stress, and hypoxia-related gene expression between high-altitude and low-altitude animals become evident (Gaur et al., 2021; Jia et al., 2016; Siques et al., 2018; Soliman et al., 2022; Tang et al., 2021; Yang et al., 2022).

Despite these differences, there is a lack of studies comparing oxidative stress, immune responses, and the expression of hypoxia-related genes in Snowy White chickens raised at high and low altitudes. This study aims to provide insights into the adaptive evolution of chicken in varying altitude environments, offering valuable information for the conservation and sustainable use of this breed.

## Materials and methods

### Ethics statement

The animal experiment was approved by the Institutional Animal Care and Use Committee of Sichuan Agricultural University (SYXK2019-187). All experiments were conducted in accordance with the guidelines provided by the Animal Welfare and Ethics Committee of Sichuan Agricultural University.

### Experimental animals, design, and management

In this study, Snowy White chickens fed at high and low altitudes were used, and the feeding locations and environments are shown in Table 1. A total of 380 low-altitude Snowy White chickens (203 days old) were raised at the Poultry Breeding Unit, Teaching and Research Facility, Ya’an Campus, Sichuan Agricultural University. Meanwhile, 550 high-altitude Snowy White chickens (203 days old) were raised at the Tibetan Chicken Breeding Base, Institute of Animal Husbandry and Veterinary Research, Academy of Agricultural and Animal Husbandry Sciences, Tibet Autonomous Region. Both high and low-altitude Snowy White chickens were fed a corn-soybean meal-based diet (Table 2). All feeding and management procedures followed the local standards outlined in the ‘Technical Specification for Breeding Lhasa White Chickens (Breed Groups)’ (DB54/T0036-2021) set by the Tibet Autonomous Region. Strict disease prevention and disinfection protocols were adhered to during the experiment. The chickens were given free access to food and water, and temperature (23 °C) and humidity (50∼70) conditions in the housing were carefully controlled in response to the chickens' needs and changes in weather.

**Table 1:** Sampling.

Chicken Breed	Feeding location	Sample size	Altitude/m	Longitude	Latitude	Oxygen partial pressure (mmHg)
Snowy white chicken	Lhasa, Tibet	24	3650	91°01′	29°26′	115.8
	Ya'an, Sichuan	24	600	102°38′	29°43′	151.8

**Table 2:** Ingredient composition and calculated nutrient content of basal diet ( % Dry matter).

Chicks (1 – 6 weeks)				Young laying hens (6 – 20 weeks)				Laying hens in the peak period (>20 weeks)			
Ingredient	Content ( %)	Nutrient	%	Ingredient	content ( %)	Nutrient	%	Ingredient	content ( %)	Nutrient	%
Corn	59.77	Metabolizable energy (ME)	12.10MJ/kg	Corn	63.00	Metabolizable energy	11.80MJ/kg	Corn	62.70	Metabolizable energy	11.09MJ/kg
Soybean meal	33.7	Crude protein	19.50	soybean meal	23.00	Crude protein	15.49	Soybean meal	26.30	Calcium	3.50
Soybean oil	2.4	Calcium	0.95	Soybean oil	3.0	Calcium	0.81	Table salt	0.30	Crude protein	16.61
Stone powder	1.3	Available phosphorous	0.4	Chaff	6.80	Available phosphorous	0.67	Calcium bicarbonate	8.50	Non-phytate phosphorus	0.35
Sodium chloride	0.25	Available phosphorous	0.4	sodium chloride	0.20	Lysine	0.73	Calcium bicarbonate	1.00	Digestible lysine	0.85
50 % choline chloride	0.1			Calcium hydrogen phosphate	3.00	Methionine+cysteine	0.64	DL-Methionine	0.10	Digestible methionine	0.35
Calcium hydrogen phosphate	1.3			Premix^2^	1.00			Choline chloride	0.10		
Methionine	0.18			**Total**	**100**			Premix^3^	1.00		
1 % premix^1^	1							**Total**	**100**		
**Total**	**100**										

Vitamin premix^1^supplied (per kg of diet: Vitamin A,9500 IU; Vitamin D3,4560 IU; Vitamin E,38 mg; Vitamin K3,3.8 mg; Vitamin B1,4 mg; Vitamin B2,7.0 mg; Vitamin B6,6.5 mg; Vitamin B12,0.03 mg; Folic acid,1.8 mg; Biotin,0.32 mg; Nicotinamide,50 mg; Cu,10 mg; Fe,65 mg; Zn,95 mg; Mn,100 mg; I,1 mg; Se,0.3 mg.

Vitamin premix^2^ supplied (per kg of diet): Vitamin A, 12000 IU; Vitamin D3, 3000 IU, Vitamin E,24 mg, vitamin K3,3 mg, Vitamin B1,12.4 mg; Niacin, 45 mg; D-pantothenic acid,12 mg; Vitamin B6, 3.6 mg; D-biotin, 0.09 mg; folate 1.2 mg; VB12,15 ug; Fe, 80 mg; Cu, 8 mg; Mu, 90 mg; Zn, 85 mg; I, 0.8 mg; Se, 0.3 mg; 98 % L-lysine, 204 mg; 50 % cholinesterase, 2.6 g; phytase, 0.1 g. Vitamin premix^3^supplied (per kg of diet): Vitamin A,10000 IU; Vitamin D3,3000 IU, Vitamin E,20 mg, Vitamin B12,5 mg; Vitamin K,3.2 mg; Folic acid,1.5 mg; Biotin, 2 mg; Pyridoxine, 8 mg; Nicotinic acid, 32.5 mg; Choline, 500 mg; Calcium pantothenate, 40 mg; Riboflavin, 8 mg; Thiamine, 1 mg; Zn, 80 mg; Fe, 80 mg; Mn, 70 mg; I,1 mg; Cu, 8 mg; Se, 0.3 mg.

**Fig. 1:**
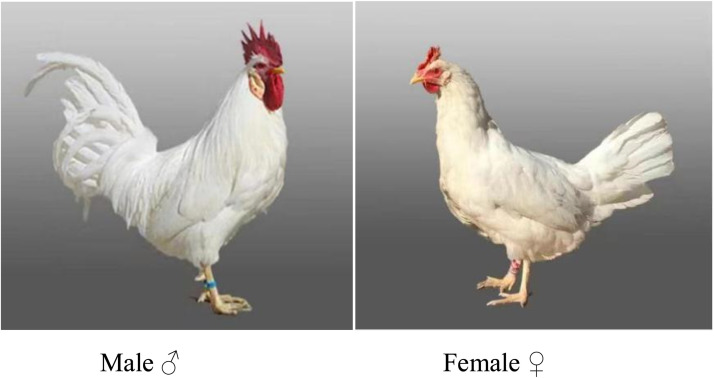
Snowy White chickens.

The composition of chick feed ingredients includes maize, soybean meal, vegetable meal, cottonseed meal, minerals, vitamins, and an anti-mold agent. For young and peak-laying hens, the feed consists of maize, soybean meal, soybean oil, calcium hydrogen phosphate, vitamin E, vitamin C, L-lactic acid hydrochloride, DL-methionine, ferrous sulfate, sodium chloride, antioxidants, preservatives, and other additives. The Snowy White Chicken is a high-altitude-adapted egg-type chicken breed jointly developed by the Institute of Animal Husbandry and Veterinary Medicine, Tibet Academy of Agricultural and Animal Sciences, and the Lhasa Poultry Germplasm Research and Promotion Center. The average age at the onset of egg laying is 178 days, with adult body weights of 1.75-2.00 kg for males and 1.30-1.45 kg for females. Their plumage is pure white, and they exhibit strong resistance to cold and hypoxia. Their images are shown in Fig. 1.

### Sample collection and procedure

Chickens were fasted for 12 hours prior to blood collection via the wing vein of both male (12) and female (12) chickens from each experimental location. A 2 mL blood sample was drawn into a sodium heparin anticoagulant vacuum blood collection tube. The tube was gently inverted and mixed several times to ensure proper mixing for routine blood analysis. In addition, 5 mL of blood was collected in additive-free vacuum blood collection tubes, allowed to stand at room temperature to enable serum precipitation. The samples were then centrifuged at 3000 rpm for 10 min, and the upper serum layer was collected and stored at -80°C for further analysis of respiratory metabolism enzymes and biochemical blood indices.

Selected chickens were humanely euthanized, and then tissues samples including the heart, liver, spleen, lungs, kidneys, and brain were immediately removed, weighed, and rinsed with PBS. For hypoxia-related gene expression analysis, approximately 1 g of each organ was divided into two 2 mL EP tubes, wrapped in tin foil, and placed in liquid nitrogen to freeze. The samples were then stored at -80°C for later analysis.

### Measurement of blood physiological parameters

Eight blood physiological indices were measured from the collected blood samples of Snowy White chickens using a veterinary automatic hematology analyzer, following the standard operational protocols. The measured indices included: total red blood cell count (RBC), hemoglobin concentration (HGB), hematocrit (HCT), mean corpuscular volume (MCV), mean corpuscular hemoglobin (MCH), mean corpuscular hemoglobin concentration (MCHC), red blood cell distribution width (RDW-SD), and red blood cell distribution width coefficient of variation (RDW-CV).

### Measurement of serum biochemical indicators

Serum metabolic enzyme activities (lactate dehydrogenase (LDH) and lactate (LA)), oxidative stress markers (malondialdehyde (MDA), total superoxide dismutase (T-SOD), catalase (CAT), total antioxidant capacity (T-AOC), and glutathione peroxidase (GSH-Px)), immunoglobulins (IgA, IgG and IgM), and inflammation-related cytokines (IL-4 and IL-6) were measured following the manufacturer’s instructions (Nanjing Jiancheng Institute of Biological Research, China (Jiangsu, China)).

### RNA extraction and real-time quantitative PCR (qRT-PCR)

Total RNA was extracted from tissue samples (heart, liver, spleen, lung, kidney, brain) using the Trizol reagent method, according to the manufacturer’s protocol. The concentration and purity of RNA were assessed using a Nanodrop 2000c spectrophotometer (Thermo Fisher Scientific, Waltham, MA, USA) based on the A260/280 absorbance ratio.

First-strand complementary DNA (cDNA) was synthesized using the PrimeScript RT kit (Takara, Dalian, China) following the manufacturer's instructions. qRT-PCR was performed on a CFX96 Real-Time System (Bio-Rad, Hercules, CA, USA) under the following cycling conditions: initial denaturation at 95°C for 3 min; 40 cycles of 95°C for 10 seconds, followed by the annealing temperature (as per Table 2, Table 3) for 20 s, then extension at 72°C for 20 seconds, with a final melting curve analysis from 65-95°C. The amplification efficiency of the target genes ranged from 95 % to 105 %. Each qRT-PCR reaction mixture (15 μL total) consisted of 6.25 μL of TB GreenTM Premix (Takara), 0.3 μL of forward and reverse primers, 1.5 μL of cDNA, and 6.65 μL of DNase/RNase-free water (Tiangen, Beijing, China). Glyceraldehyde-3-phosphate dehydrogenase (GAPDH) was used as the internal reference gene for normalization of gene expression. The gene expression was quantified using the 2^-ΔΔCt^ method according to our previous study (Cui et al., 2020). Primer sequences were designed using Primer 5 software based on the coding sequences of the target genes (Table 3).

**Table 3:** Primers used for qPCR.

Gene	Sequence (5’-3’)	Annealing Temperature (°C)	Accession Number
*GAPDH*	*F: CGTCCTCTCTGGCAAAGTCC*	60.30	NM_204305.2
	*R: ACAGTGCCCTTGAAGTGTCC*		
*HIF-1A*	*F: AATGCCGATCCTGCACTCAA*	58.03	NM_001396327.1
	*R: CATCAGAAGGGCTGGTTGGT*		
*EPAS1*	*F: TTATTGCCGTGGTGAC*	58.65	NM_204807.3
	*R: CTTGCTGTCCAGAGGG*		
*EGLN1*	*F: CCCAGGCAATGGAACAGGAT*	56.10	XM_015284393.4
	*R: GCCTCCACTTACCTTGGCAT*		
*VEGFA*	*F: CCCAACGAAGTTATCAAAT*	58.3	NM_001110355.2
	*R: CAACCCGCACATCTCA*		
*LDHB*	*F: CACAGCCAACTCCAAGA*	58.47	NM_204177.3
	*R: AACTCCGCTCCAAACA*		
*EPO*	*F: ACCCCGGCGTCAGCTTCA*	57.32	XM_046898341.1
	R: CGGACTTTGCCACGGAGGA		

### Statistical analysis

The experimental data were statistically analyzed using Excel. For comparisons between two independent sample groups, an independent samples t-test was performed using SPSS 23. For the analysis of hypoxia-related gene expression, one-way analysis of variance (ANOVA) was conducted in SPSS 23. All results are presented as mean ± standard deviation, with *P* < 0.05 indicating statistical significance. Data visualization was performed using GraphPad Prism 9.5.1.

## Results

### Serum metabolic profile of snowy white chickens at high and low altitudes

**Fig. 2:**
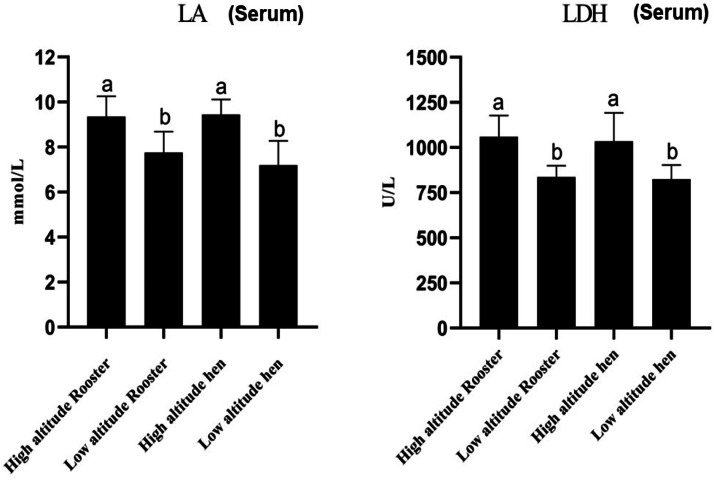
Comparison of serum metabolic indices in Snowy white chickens raised at high and low altitudes. The Fig. illustrates the concentrations of lactic acid (LA) and lactate dehydrogenase (LDH) in the serum of chickens reared under high-altitude and low-altitude conditions. These metabolic markers are indicative of anaerobic respiration activity. Elevated levels reflect increased glycolytic metabolism, commonly associated with hypoxic stress. Data are presented as mean ± standard deviation (SD). Different lowercase letters (a, b) denote statistically significant differences between groups (*P* < 0.05).

To investigate the changes in respiratory metabolism of Snowy White chickens at different altitudes, we measured the serum lactate (LA) and lactate dehydrogenase (LDH) levels of chickens at both high and low altitudes (Fig. 2). The results indicated that serum lactate and lactate dehydrogenase levels were significantly higher in both male and female Snowy White chickens at high altitude compared to their counterparts at low altitude (*P* < 0.05).

### Blood physiological indices of snowy white chickens at high and low altitudes

In this study, key hematological parameters demonstrated significant altitudinal differences (Table 4). The results showed that the blood physiological indices of the Snowy White chickens exhibited significant differences between high- and low-altitude environments (Table 4). The red blood cell (RBC) count was significantly higher in high-altitude roosters (4.47 ± 0.29 × 10⁶/μL) and hens (3.27 ± 0.36 × 10⁶/μL) compared to their low-altitude counterparts (3.14 ± 0.33 × 10⁶/μL for roosters and 2.34 ± 0.22 × 10⁶/μL for hens, *P* < 0.05). Hemoglobin (HGB) levels followed a similar pattern, being significantly greater in high-altitude chickens (243.83 ± 18.13 g/L in roosters and 257.19 ± 34.00 g/L in hens) than in low-altitude chickens (200.75 ± 19.42 g/L and 139.94 ± 10.87 g/L, respectively, *P* < 0.05). The hematocrit (HCT) values were also markedly increased at high altitude, thus, 54.47 ± 3.26 % in roosters and 39.51 ± 5.29 % in hens, compared to 37.60 ± 3.86 % and 28.76 ± 2.08 % in their low-altitude counterparts (*P* < 0.05). However, mean corpuscular volume (MCV) was significantly higher in low-altitude chickens, especially in roosters (119.86 ± 3.34 fL and 88.95 ± 2.78 fL; *P* < 0.05). Similarly, the standard deviation (RDW-SD) of erythrocyte distribution width was higher in low-altitude birds (64.59 ± 7.36 in roosters, 45.48 ± 3.00 in hens) than in those raised at high altitude (50.93 ± 4.28 and 38.33 ± 2.02, respectively; *P* < 0.05). The coefficient of variation (RDW-CV) was also elevated in low-altitude roosters (11.38 ± 0.56 %) compared to high-altitude roosters (10.28 ± 1.16 %), while hens showed the opposite trend, with low-altitude hens recording a lower CV (9.18 ± 0.61 %) than high-altitude hens (11.01 ± 0.77 %) (*P* < 0.05).

**Table 4:** Blood physiological indices of Snowy White chickens raised at high and low altitudes.

Indicators	Rooster		Hen	
	High altitude	Low altitude	High altitude	Low altitude
RBC	4.47±0.29^a^	3.14±0.33^b^	3.27±0.36^b^	2.34±0.22^c^
HGB	243.83±18.13^a^	200.75±19.42^b^	257.19±34.00^a^	139.94±10.87^c^
HCT	54.47±3.26^a^	37.60±3.86^b^	39.51±5.29^b^	28.76±2.08^c^
MCV	88.95±2.78^b^	119.86±3.34^a^	121.17±3.74^a^	123.00±4.36^a^
MCH	78.49±3.16^a^	64.03±1.84^b^	78.78±3.32^a^	59.85±1.89^c^
MCHC	575.33±18.15^b^	534.50±18.06^c^	650.94±20.31^a^	486.25±6.63^d^
SD/RDW-SD	50.93±4.28^b^	64.59±7.36^a^	38.33±2.02^d^	45.48±3.00^c^
CV/RDW-CV	10.28±1.16^b^	11.38±0.56^a^	11.01±0.77^ab^	9.18±0.61^c^

**Note:** All results are presented as mean ± standard deviation. Different superscript letters within the same row indicate significant differences.

### Oxidative stress indicators in snowy white chickens at high and low altitudes

In this study, the antioxidant capacity in the serum of Snowy White chickens from different altitudes was evaluated (Table 5). Oxidative stress-related parameters showed significant altitude-dependent differences (Table 5). The malondialdehyde (MDA) level, an indicator of lipid peroxidation, was highest in high-altitude hens (7.29 ± 0.65 nmol/mL) and lowest in low-altitude hens (6.01 ± 0.30 nmol/mL, *P* < 0.05). Roosters at high and low altitudes recorded intermediate MDA levels (6.71 ± 0.72 nmol/mL and 6.35 ± 0.83 nmol/mL, respectively). Total antioxidant capacity (T-AOC) was significantly higher in low-altitude roosters (10.74 ± 1.04 U/mL) and hens (9.03 ± 0.83 U/mL) compared to high-altitude roosters (8.41 ± 0.66 U/mL) and hens (7.92 ± 0.57 U/mL) (*P* < 0.05). Similarly, total superoxide dismutase (T-SOD) activity was increased in low-altitude birds (162.59 ± 5.10 U/mL for roosters and 158.92 ± 5.49 U/mL for hens) compared to high-altitude chickens (152.30 ± 5.11 U/mL and 150.19 ± 7.33 U/mL, respectively; *P* < 0.05). While GSH-px and CAT values did not differ significantly among most groups, GSH-px was notably higher in low-altitude hens (2518.60 ± 222.38 U/mL) compared to high-altitude hens (2408.14 ± 291.93 U/mL) (*P* < 0.05).

**Table 5:** Oxidative stress parameters of Snowy White chickens raised at high and low altitudes.

Measurement indicators	Rooster		Hen	
	High altitude	Low altitude	High altitude	Low altitude
MDA (nmol/mL)	6.71±0.72^b^	6.35±0.83^bc^	7.29±0.65^a^	6.01±0.30^c^
CAT(U/mL)	5.60±0.45	5.47±0.61	5.54±0.49	5.58±0.42
GSH-px(U/mL)	2196.51±176.93^b^	2279.07±314.77^ab^	2408.14±291.93^ab^	2518.60±222.38^a^
T-AOC(U/mL)	8.41±0.66^c^	10.74±1.04^a^	7.92±0.57^c^	9.03±0.83^b^
T-SOD(U/mL)	152.30±5.11^b^	162.59±5.10^a^	150.19±7.33^b^	158.92±5.49^a^

**Note:** All results are presented as mean ± standard deviation. Different superscript letters within the same row indicate significant differences.

### Immune Indices in Snowy White Chickens at High and Low Altitudes

Immune-related indices showed that Snowy White chickens raised at low altitudes had significantly stronger humoral immunity (Table 6). IgM levels were highest in low-altitude roosters (571.20 ± 45.54 ng/mL), followed by low-altitude hens (488.84 ± 56.00 ng/mL), with significantly lower values observed in high-altitude roosters (465.24 ± 59.82 ng/mL) and hens (438.81 ± 42.80 ng/mL; *P* < 0.05). Similarly, IgG concentrations were elevated in low-altitude chickens 29.06 ± 4.02 μg/mL in roosters and 27.98 ± 3.21 μg/mL in hens compared to 22.16 ± 1.92 μg/mL and 22.49 ± 2.52 μg/mL in high-altitude roosters and hens, respectively (*P* < 0.05). No significant differences were observed in IgA, IL-4, or IL-6 levels between the groups (*P* > 0.05), suggesting that the most prominent adaptive differences occur in the IgM and IgG responses.

**Table 6:** Immune parameters of Snowy White chickens raised at high and low altitudes.

Indicator	Rooster		Hen	
	High altitude	Low altitude	High altitude	Low altitude
lgA (ng/mL)	965.86±161.94	976.90±104.94	962.47±133.87	975.03±119.78
lgM (ng/mL)	465.24±59.82^bc^	571.20±45.54^a^	438.81±42.80^c^	488.84±56.00^b^
lgG (ug/mL)	22.16±1.92^b^	29.06±4.02^a^	22.49±2.52^b^	27.98±3.21^a^
IL-6 (ng/L)	3.16±0.45	3.30±0.59	3.41±0.37	3.48±0.50
IL-4 (ng/L)	14.80±2.08	15.01±2.41	15.22±2.27	14.17±1.26

**Note:** All results are presented as mean ± standard deviation. Different superscript letters within the same row indicate significant differences.

### Expression of hypoxia-related genes in snowy white chickens at high and low altitudes

**Fig. 3:**
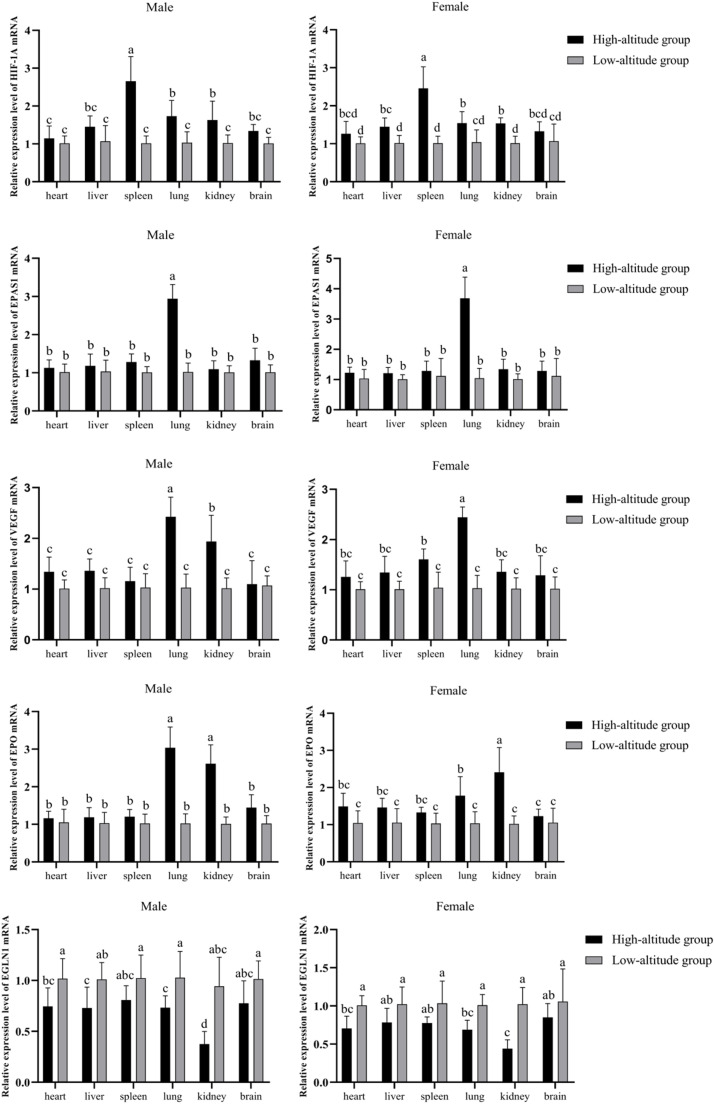
Expression of hypoxia-related genes in snowy white chickens kept at high and low altitudes. L=Low altitude group; H = High altitude group. ^a,b,c,d^ indicates significant difference (*P*<0.05).

The expression profiles of hypoxia-responsive genes were distinctly altered in chickens raised at high altitude (Fig. 3). HIF-1A expression was significantly upregulated in the spleen, lungs, and kidneys of high-altitude chickens of both sexes (*P* < 0.05), with the spleen showing the highest expression in hens. In hens, this increase was also observed in the liver (P < 0.05), with the spleen showing the highest HIF-1A levels. EPAS1 expression was markedly increased in the lungs, particularly in high-altitude roosters (*P* < 0.05). High-altitude conditions also significantly increased VEGF expression in the lungs and kidneys of roosters (P < 0.05), while in hens, the lungs and spleen showed the highest levels (*P* < 0.05). In both roosters and hens, VEGF levels were highest in the lungs. Similarly, the expression of EPO was significantly higher in the lungs and kidneys of high-altitude chickens (*P* < 0.05), with the highest levels found in the lungs of roosters and the kidneys of hens. Furthermore, EGLN1, which negatively regulates HIF signaling, was also significantly increased in the heart, lungs, and kidneys of high-altitude chickens compared to low-altitude controls (*P* < 0.05), indicating a possible feedback adaptation. High-altitude roosters exhibited higher EGLN1 expression than their low-altitude counterparts across all three tissues.

## Discussion

High-altitude environments are characterized by reduced atmospheric pressure and oxygen levels, which significantly affect animal physiology (Soliman et al., 2022). In such conditions, animals have evolved various adaptive mechanisms to cope with these environmental challenges. One of the primary responses to high-altitude hypoxia is the activation of anaerobic respiration when oxygen availability is insufficient. This adaptation triggers changes in respiratory enzyme activities, enabling animals to optimize oxygen utilization for energy production (Soliman et al., 2022). Oxygen is essential for the metabolism and functions of mammals. However, in certain environments, such as subterranean burrows, high altitudes, or the deep ocean, oxygen availability is limited, which can impose hypoxic stress and lead to oxidative damage in organisms living in these habitats (Li et al., 2021). To survive in these conditions, organisms have developed specialized strategies to adapt to hypoxia, including physiological adjustments, regulation of gene expression, and genetic mutations (Li et al., 2021).

This study revealed that Snowy White chickens raised at low altitudes exhibited significantly lower levels of serum lactate and lactate dehydrogenase compared to those at high altitude. These findings suggest that the hypoxic conditions at high altitude induce an increase in anaerobic metabolism, highlighting a physiological adaptation to low oxygen availability. The adaptation of blood physiology to varying altitudes has been well documented, with animals at higher altitudes typically exhibiting higher numbers of red blood cells and hemoglobin content to enhance oxygen transport. However, this can increase blood viscosity, leading to higher blood pressure (Okur et al., 2022; Yu et al., 2024; Zhu et al., 2020). In this study, we observed that Snowy White chickens raised at low altitudes had significantly lower levels of red blood cells, hemoglobin, and hematocrit compared to those raised at high altitudes, reflecting a reduced need for oxygen-carrying capacity at lower altitudes. However, the mean corpuscular volume of red blood cells was significantly higher in low-altitude chickens, suggesting a compensatory mechanism for lower red blood cell count. This adaptation minimizes the oxygen-carrying capacity per red blood cell, which is typical in low-altitude environments. Exposure to high altitudes, where oxygen pressure is lower, can lead to oxidative and reductive stress, promoting the generation of reactive oxygen and nitrogen species (RONS), which in turn cause oxidative damage to lipids, proteins, and DNA. The intensity of oxidative stress is directly related to the altitude level (Dosek et al., 2007; Mallet et al., 2023). Various systems that generate RONS are activated during high-altitude exposure, including the mitochondrial electron transport chain, xanthine oxidase, and nitric oxide synthase (Pena et al., 2022). High-altitude conditions also seem to impair both enzymatic and non-enzymatic antioxidant defenses. Increasing the intake of antioxidant vitamins may help mitigate oxidative damage caused by high-altitude exposure (Pena et al., 2022). Our study found that Snowy White chickens at low altitudes exhibited higher levels of antioxidant enzymes, including T-AOC and T-SOD, compared to those at high altitudes. This suggests an adaptive increase in antioxidant capacity at lower altitudes, which aligns with previous findings in other species (Soliman et al., 2022).

The immune response to altitude-induced stress was investigated by measuring the immunoglobulin levels. We found that low-altitude chickens had significantly higher levels of IgM and IgG compared to those at high altitudes. This supports the hypothesis that altitude influences immune function and that chickens raised at low altitudes are better equipped to handle environmental stressors (Wang, et al., 2019). In addition, the observed differences in IgM levels between roosters and hens at low altitude could be attributed to increased immune system activation due to higher activity levels in roosters, which could enhance immune response.

Hypoxia-related genes, including *EGLN1, HIF-1A, EPAS1, VEGF,* and *EPO*, play crucial roles in adapting to low oxygen environments. This study revealed that high-altitude chickens exhibited markedly elevated HIF-1A expression, especially in the spleen, lungs, and kidneys, suggesting a strong adaptive response to hypoxic conditions. HIF-1A activation facilitates adaptive mechanisms such as angiogenesis and erythropoiesis, which improve tissue oxygenation (Vageli et al., 2024; Wang, et al., 2019, 2024). Moreover, EPO expression was elevated in the lungs and kidneys of high-altitude chickens, indicating a compensatory adaptation to enhance oxygen transport in these organs. This observation aligns with studies in other high-altitude species, such as yaks, where increased EPO levels promote erythropoiesis and improve oxygen delivery (Yang et al., 2023). In this present study, the physiological adaptations observed in high-altitude Snowy White chickens were mediated by hypoxia-inducible signaling cascades. Central to this adaptation is the stabilization of hypoxia-inducible factor 1-alpha (HIF-1α), a transcription factor that becomes active under low oxygen tension due to inhibited prolyl hydroxylase activity (such as EGLN1). Once stabilized, HIF-1α translocates to the nucleus and dimerizes with HIF-1β, triggering transcription of genes such as *EPAS1, VEGF,* and *EPO.* Furthermore, *EPAS1* supports cellular oxygen sensing, VEGF enhances capillary density and tissue perfusion, while EPO promotes erythropoiesis in the bone marrow, thereby increasing oxygen transport capacity. These molecular events are essential for maintaining cellular bioenergetics and systemic oxygen homeostasis in hypoxic environments. Concurrently, oxidative stress response genes are modulated to counteract ROS accumulation, which is a byproduct of mitochondrial respiration under hypoxia. The observed elevation in T-SOD and T-AOC suggests a coordinated antioxidant response that preserves tissue integrity. Collectively, these molecular and enzymatic processes form an integrated hypoxia-response network that underpins the survival and productivity of Snowy White chickens at high altitudes.

## Conclusions

This study comprehensively demonstrated that Snowy White chickens raised at high altitudes undergo distinct physiological and molecular adaptations that enable them to maintain homeostasis under chronic hypoxic stress. Chickens at high altitude showed enhanced anaerobic metabolism, as evidenced by increased serum lactate and lactate dehydrogenase levels, and elevated oxygen-carrying capacity, reflected by higher red blood cell count, hemoglobin concentration, and hematocrit compared to their low-altitude counterparts. In contrast, chickens reared at low altitudes exhibited stronger antioxidant defense and enhanced immune responses, demonstrated by significantly higher levels of T-AOC, T-SOD, IgM, and IgG, and lower levels of malondialdehyde, a marker of lipid peroxidation. Furthermore, hypoxia-related gene expression patterns confirmed molecular adaptation, with high-altitude chickens showing upregulation of *HIF-1A, EPAS1, VEGF*, and *EPO*, and downregulation of *EGLN1* in key tissues including the heart, lungs, and kidneys. Together, these findings provide valuable insights into the altitude-driven physiological and genetic plasticity of Snowy White chickens and reveal how environmental hypoxia shapes their metabolic, hematological, and molecular profiles. This study deepens our understanding of avian adaptation to high-altitude environments as well as offers practical implications for breeding strategies, genetic selection, and conservation efforts aimed at optimizing poultry health and productivity across diverse altitudinal landscapes.

## Ethics approval and consent to participate

The animal experimental procedures were approved by the Institutional Animal Care and Use Committee of Sichuan Agricultural University, China (2022. 12. 06. Certification No. SYXK2019-187), and all the experiments were conducted in accordance with the guidelines provided by the Sichuan Agricultural University Laboratory Animal Welfare and Ethics.

## Declaration of competing interest

The authors declare that they have no known competing financial interests or personal relationships that could have appeared to influence the work reported in this paper.
